# Retraction: Functional mechanism of AMPK activation in mitochondrial regeneration of rat peritoneal macrophages mediated by uremic serum

**DOI:** 10.1371/journal.pone.0306659

**Published:** 2024-08-02

**Authors:** The PLOS ONE Editors

After this article [1] was published, concerns were raised regarding the similarity to previous works [2–4] by different authors and that were not cited in [1].

Specifically, the overall concept, objectives, study design, and methodology of this article [1] are similar to work reported in previously published Master’s theses [2,3] and a published article [4]. The experimental design and results reported in all figures and tables of [1] appear very similar to those previously reported in [2,3], though the individual data values reported are not identical.

In response to these concerns, one author commented that the idea to examine the interplay between AMPK activation and macrophage polarization in the rat model of chronic kidney disease followed logically from findings reported in other studies, and that their team conceived the idea, conducted the experiments and collected the data. The author provided underlying data for Figs 1, 3, 4, 5, and 6, and raw RT-PCR data for Figs 3, 4, 5, and 6. Editorial evaluation noted discrepancies between these data and the underlying data deposited in Dryad at the time of publication; a different version of the dataset was subsequently provided.

Overall, the comments and data received from the author did not resolve PLOS’ concerns about the data provided for [1] and the high degree of similarity between the research reported in [1] and [2–4].

Additionally, the article [1] reported the use of ether for euthanasia without specific scientific justification for its use, which is not in adherence with *PLOS ONE*’s submission guidelines in effect at the time of submission. The corresponding author indicated there was an error in the reported methodology, and that the method of euthanasia used was CO_2_ asphyxiation.

In light of the unresolved concerns about the data and about similarities between this article [1] and the previous works [2–4], the *PLOS ONE* Editors retract this article.

DC, KZ, XLiang, MW, HZ, and RZ either did not reply directly or could not be reached. XLiu did not reply directly from their own email address to comment on the decision.

The retracted article [1] was removed from the *PLOS ONE* website at the time of retraction due to the similarities to [2–4]. The article’s Copyright and Data Availability statements were also updated at the time of retraction, and the removed contents are no longer offered under the Creative Commons Attribution License.
