# Correction: Retraction: Functional mechanism of AMPK activation in mitochondrial regeneration of rat peritoneal macrophages mediated by uremic serum

**DOI:** 10.1371/journal.pone.0309246

**Published:** 2024-08-15

**Authors:** The PLOS ONE Editors

The *PLOS ONE* Editors issue this notice to correct an error in the retraction notice [1] for this article [2]. Contrary to the statement in the retraction notice, the article was not removed from the *PLOS ONE* website at the time of retraction but instead was removed on August 9, 2024. The article’s Copyright and Data Availability statements have now also been updated, and the removed contents are no longer offered under the Creative Commons Attribution License.

The publisher apologizes for the error.
